# Adapting Axelrod’s cultural dissemination model for simulating peer effects

**DOI:** 10.1016/j.mex.2016.11.003

**Published:** 2016-11-21

**Authors:** Christian Hofer, Gernot Lechner, Thomas Brudermann, Manfred Füllsack

**Affiliations:** University of Graz, Institute of Systems Sciences, Innovation and Sustainability Research, Austria

**Keywords:** Agent-based modeling with incomplete empirical data, Cultural dissemination, Peer effects, Agent-based modeling, Incomplete data, Diffusion processes

## Abstract

We present a generic method for considering incomplete but gradually expandable sociological data in agent-based modeling based on the classic model of cultural dissemination by Axelrod. Our method extension was inspired by research on the diffusion of citizen photovoltaic initiatives, i.e. by initiatives in which citizens collectively invest in photovoltaic plants and share the profits. Owing to the absence of empirical interaction parameters, the Axelrod model was used as basis for considering peer effects with contrived interaction data that can be updated from empirical surveys later on. The Axelrod model was extended to cover the following additional features:

• Consideration of empirical social science data for concrete social interaction.
• Development of a variable and fine-tunable interaction function for agents.
• Deployment of a generic procedure for modeling peer effects in agent-based models.

## Graphical abstract

**Figure:**
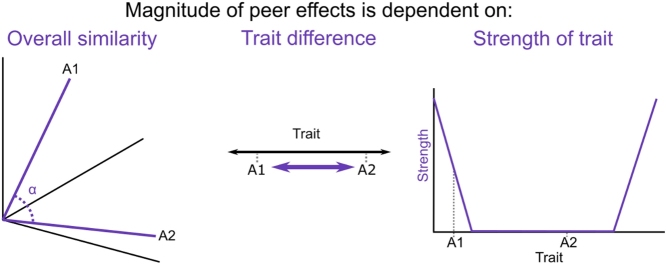

## Method details

In this paper we describe a generic way of considering incomplete but gradually expandable sociological data in agent-based modeling. In extension to Axelrod’s model of cultural dissemination [1], we developed a generic procedure for feeding the model with data that can be completely or partially contrived at first, and can be easily updated later on when additional information becomes available. Our aim was to conceive the model in a way that allows for informative utilization even without exact data. We applied the method in simulating the bottom-up diffusion of photovoltaic citizen power plants in Austria [2], [3]. The focus was on complementing individual traits of respondents from an empirical survey with missing information on peer effects. In order to allow for utmost flexible consideration of impact strength and decision uncertainties, a generic function for varying relevant parameters is proposed. Background information regarding the application of the method described in this specific case, and a brief description of the original Axelrod model are provided in the section ‘Additional information’ towards the end of the paper.^1^

### Preprocessing of data

In the social sciences, data is often gathered in the form of a questionnaire where respondents are asked to report upon their attitudes towards a specific topic. Likert scales, in which individual items can be rated using a scale ranging from, e.g., “strongly agree” to “strongly disagree”, are used for recording purposes. It is our intention here to use the data collected in such a manner to describe the characteristics of agents in agent-based simulation models. Note that within the scope of this paper, we refer to the characteristics of agents as “traits”. Traits may include attitudes, behaviors and perceptions, or other characteristics used to describe the participants of empirical studies. In our application of the method modification, these traits relate to characteristics associated with the adoption of PV; however, the procedure described is quite generic and may be used to model peer effects in other domains as well.

The granularity of possible responses in Likert scales can vary. The most common scales have between 4 and 7 points. To neutralize this source of variation, we normalize Likert scales values so that they conform to a scale ranging from −1 to +1. The value −1 indicates strong discordance with the respective trait, and the value +1 indicates strong accordance with the respective trait. Such normalization not only allows for inclusion of data from different Likert scales, it also facilitates subsequent mathematical operations.

When using such data for agent-based simulation models we need to consider that, among other things, the data is typically not normally distributed. Data tends to follow an asymmetric distribution, e.g. revealing a large number of respondents who strongly agree, and a very small number of respondents who disagree. In addition, not all items/concepts may be relevant for the research question at hand. Thus, prior to undertaking further processing of the data, we suggest first that those traits offering high information gain with respect to the research question be identified. In the present case, for example, this entails identifying those traits which contribute to explaining whether agents participate in citizen power plants or not.

Where no suitable empirical interaction parameters are available, the respective parameters for agent interaction need to be derived from the relevant literature and from previous studies. The way of considering agent interaction in our model is inspired by the theory of innovation diffusion [4], [5], by the various suggestions on modeling the adoption of new technologies [6], by insights from social psychology [7], and by Axelrod’s original model [1], which previously has been modified and extended, e.g. to investigate the effect of mass media on a social system [8] or to investigate the role of opinion leaders in the collective behavior of a society [9].

### Agent interaction

After the initialization of the agents with the normalized empirical data as described above, agents start interacting with each other as in the original Axelrod model. In every simulation step, two agents A1 and A2 are randomly chosen, and A1 influences A2. In the original model, the influence is given by the probability of interaction, corresponding to the fraction of shared components (or overlap) of the cultural vectors of A1 and A2. This fraction should be greater than 0 and less than 1 for interaction to occur. Also, as a result of the interaction, A2 adopts one trait value from A1 (see Section ‘Additional information’ for details). However, in our modification, the type and degree of influence are modeled differently. Every element of the trait vector of agent A2 is influenced, and the magnitude of influence is dependent on the following three factors:

• Factor 1: Overall similarity of agents’ traits, modeled by cosine similarity over all traits.
• Factor 2: Differences between the interacting agents with respect to a specific trait.
• Factor 3: Degree of accordance/discordance regarding a trait.

**(1) Overall similarity of traits**

We assume that the similarity of two agents’ trait vectors affects the degree of influence they exert on each other. The more similar two agents are, the higher the degree of mutual influence. This assumption is in line with insights from social psychology; normative social influence is stronger when exerted by peers who have similar attitudes [7], [10].

To assess the similarity of two agents, we calculated the *cosine similarity* (CS) of a transform of the agents’ traits vectors. CS values range from −1 to +1, where −1 indicates total opposition, and +1 indicates total alignment. CS indicates the similarity between the two vectors of an inner product space by measuring the cosine of the angle between them. CS indicates the similarity of the inner product space of two vectors by measuring the cosine of the angle between them.

**Fig. 1:**
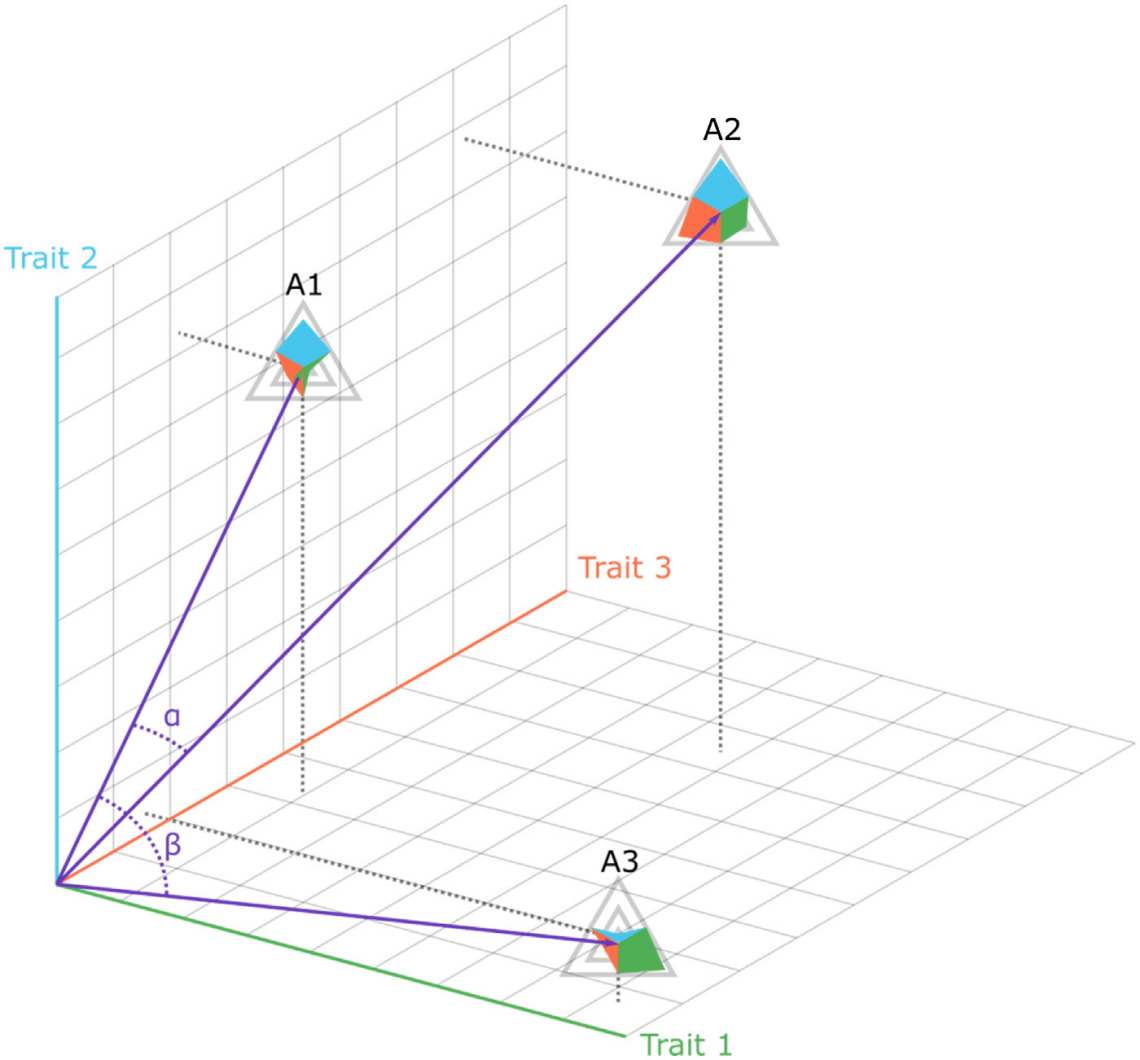
Cosine similarities based on angles α and β in a 3-dimensional example.

Three agents with trait vectors of length 3 are illustrated in Fig. 1. Agent A2’s traits are more aligned with agent A1’s traits (=higher CS value) than with A3’s traits (=smaller CS value).

Without the normalization of the agent vector range between −1 and +1, the calculations would have led to the CS values being too small as the vectors would only occupy part of the n-dimensional space. To aid further processing we scale the calculated CS to an interval [0,1]. The resulting value is defined as the *trait difference* (*td*), and ranges from 0 (for total alignment) to 1 (for total opposition).

The overall similarity of agents’ traits is expressed as follows:

(1) $${\left(1-td\right)}^{imp_{td}}$$

A decrease (increase) in *td* leads to an increase (decrease) in the impact of the influencing agent on the affected agent. This impact can be observed in Eq. (1), whereby a rise in the parameter imp_td_ induces a decreasing effect on the other agent. This may be viewed as reflecting the impact of the persuasiveness of an agent as a function of his/her own and other agents’ entire value systems.

**(2) Differences among agents with respect to a specific trait**

In contrast to the trait similarity modeled above, this measure illustrates only the difference of values regarding the current trait. The more similar, or less different, two agents are regarding a specific trait, the stronger the influence.

**Fig. 2:**
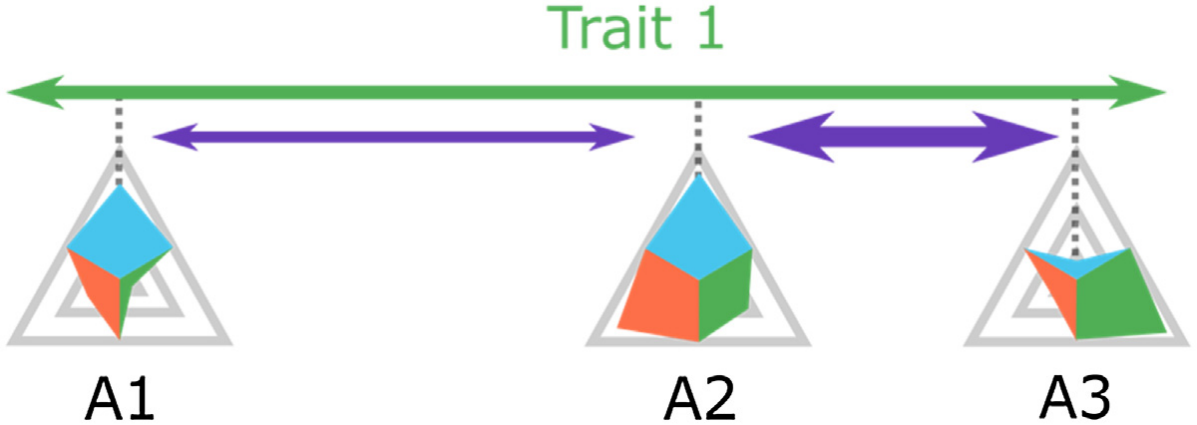
Exemplary positions of agents with respect to a specific trait (Trait 1) and the respective influence. Larger distance entails weaker influence.

In the example illustrated in Fig. 2, three agents are shown. Given their respective positions regarding trait 1, the influence exerted upon agent A2 by agent A3 will be stronger than that exerted by agent A1.

Mathematically, the difference among two agents with respect to a specific trait is expressed as follows:

(2) $$\left(1-\frac{\left|\left(x_{infl}-x_{aff}\right)\right|}{2}\right)$$

The term reflects the assumption that a higher difference in two agents’ traits implies lower influence. Here, the absolute value of the difference between the x-axis value of the influencing agent (*x_infl_*) and the x-axis value of the affected agent (*x_aff_*) is computed. As the maximum difference between +1 and −1 is 2, normalization is achieved by dividing the respective value by 2. In order to make sure that the impact is highest when the agents are on the same x-position and lowest when they are on x-positions of −1 and +1, respectively, the result of the former calculation is subtracted from 1.

**(3) Degree of accordance/discordance regarding a trait (strength of the trait)**

The third component modeled is the absolute position of a trait. We assume that agents with strong positions – that is, a strong accordance or discordance regarding a certain trait – are less likely to change this position. This complies with the “confirmation bias” effect found in psychological [11], i.e. the common observation that once somebody takes a clear position, they will not abandon it easily.

**Fig. 3:**
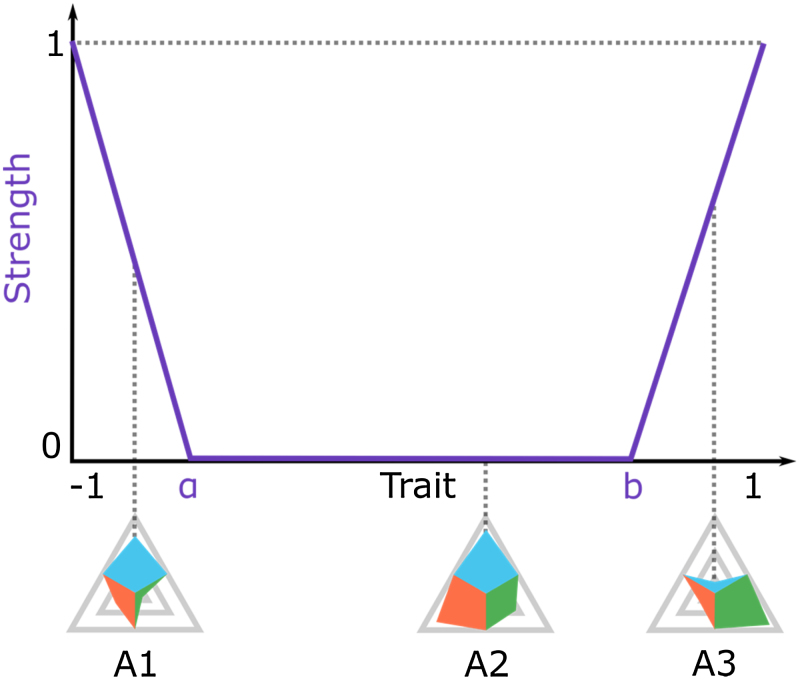
Strength of the trait function.

Following the assumptions and simplifications provided above, an agent with a strong trait value (close to −1 or +1) has more influence on other agents than an agent with a weaker trait (closer to 0). In Fig. 3, agent A3 would have a greater influence on A2 than A1. In contrast, A2’s position is relatively weak, and therefore this agent’s influence on A1 and A3 is relatively small. In the case of interaction between A1 and A3, the impact of A1 on A3 is less than the impact of A3 on A1. However, in order to quantify the impact of the agents’ interaction, a more complex function is necessary.

In order to keep the model comprehensible we introduce a simple bathtub function to model agent properties. The respective trait is mapped on the x-axis, and its strength, on the y-axis. This function is defined as follows:

(3) $$\begin{array}{c} f(x)=\frac{\left(a-x\right)}{\left(a+1\right)}if-1\leq x<a\\ f(x)=0\;if\;a\leq x\leq b\\ f(x)=\frac{x-b}{1-b}if\;b<x\leq 1 \end{array}$$

The relation between the strength (y-value [0,1]) and the current value concerning the trait (x-value [–1,1]) is therefore manifested in a piecewise linear function. Note that this procedure allows for a further rise in the complexity of the function, since piecewise linear functions may include an unrestricted number of linear functions.

The x-coordinate quantifies the agent’s level of accordance with a specific trait, with −1 reflecting total discordance, and +1 total accordance. The y-coordinate indicates the respective strength of each agent position, with 0 indicating a weak position, and 1 a strong position. Fig. 3 illustrates this function graphically.

The degree of accordance/discordance with respect to a specific trait is included in the interaction function by the following term:

(4) $${\left(1+\left(y_{infl}-y_{aff}\right)\right)}^{imp_{y}}$$

Eq. (4) thus represents the strength of the trait of the agents concerned with respect to an individual topic. The value on the y-axis of the influencing agent is *y_infl_*, while the value of the affected agent is *y_aff_*. We assume that an agent with a stronger trait has higher influence than an agent with a weaker trait. Naturally, the difference between the agents’ y-axis-values can be seen as the driver of impact: if the influencing agent exceeds the affected agent regarding his/her persuasiveness (*y_infl_* < *y_aff_*), the difference (*y_infl_* − *y_aff_*) is positive and the result of the total term is greater than 1. Where the contrary is the case, and the affected agent is on a stronger position, the difference is negative, resulting in a value of less than 1, and thus less impact is exerted compared to the former case. There is an additional control parameter imp_y_ which moderates the impact of the term on the total formula.

### Integration of interaction parameters

When agents interact, the affected agent moves towards the influencing agent’s trait. The magnitude of the impact is calculated under consideration of the three different factors discussed above:

(5) $$\frac{\left({\left(1-td\right)}^{imp_{td}}\right)*\left(1-\frac{\left|\left(x_{infl}-x_{aff}\right)\right|}{2}\right)*{\left(1+\left(y_{infl}-y_{aff}\right)\right)}^{imp_{y}}}{imp_{d}}$$

In addition to the three terms already discussed, the constant imp_d_ determines the strength of the impact of the influencing agent on the affected agent and ensures that the resulting values remain within the appropriate range. In general, all values *imp_d_* > = 1 can be used. However, in the course of the experiments a value greater than or equal to 10 turned out to be most appropriate.

### Validation and simulation results

The modifications outlined above allow for the integration of empirical data into the original Axelrod model (of cultural dissemination). However, the nature of agent interaction is now more realistic in that here it may also occur in the absence of completely identical traits, does not lead to immediate trait ‘flipping’, and allows for (step-wise) convergence.

We conducted simulations using both empirical and artificial data. Empirical data originated from a study on a citizen participation initiative in photovoltaics in Austria (n = 236). The sample included participants as well as non-participants who were aware of the initiatives, and data was collected in order to identify the drivers behind participation (e.g. attitudes with respect to renewable energy, financial considerations, etc.). In this sample, traits were rather homogeneous, i.e. most respondents were positive/negative with regards to the same traits (but to different degrees). Artificial data was generated in order to simulate more heterogeneous positions.

In both the empirical and the artificial case, the following interaction parameters were chosen: *a* = −0.2, *b* = 0.2, *imp_d_* = 10, *imp_td_* = 2, *imp_y_* = 2; 25,000 ticks per simulation run.

**Fig. 4:**
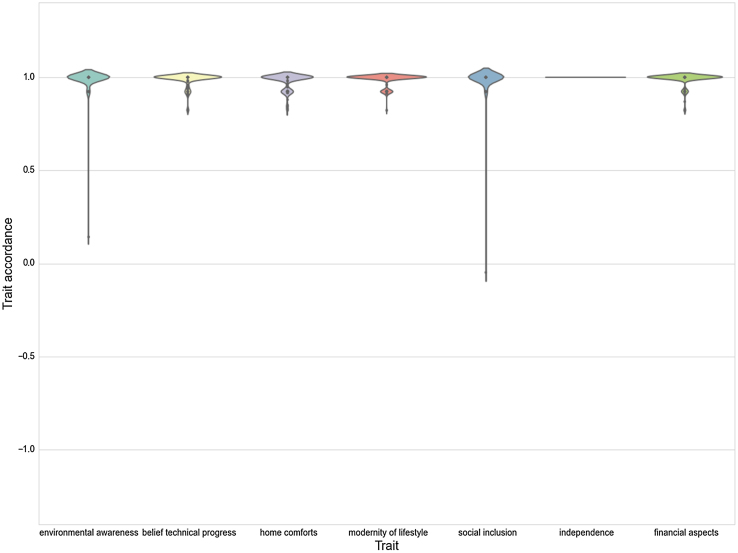
Violin plot of empirical data after simulation runs. Trait accordance for all traits converge around high positive values.

As in the original model, agent convergence was observed. In the very homogeneous empirical setting convergence was reached rather quickly, and all simulation runs produced strong convergence with respect to traits. The simulation model thus behaves as expected, since the empirical data comprises an overall majority of traits with high positive values (strong accordance with trait), and converge around high positive values. This is shown in Fig. 4 in the violin plot, which is a combination of box plot and kernel density plot. The trait distribution is visualized on the y-axis and the different traits are shown on the x-axis.

**Fig. 5:**
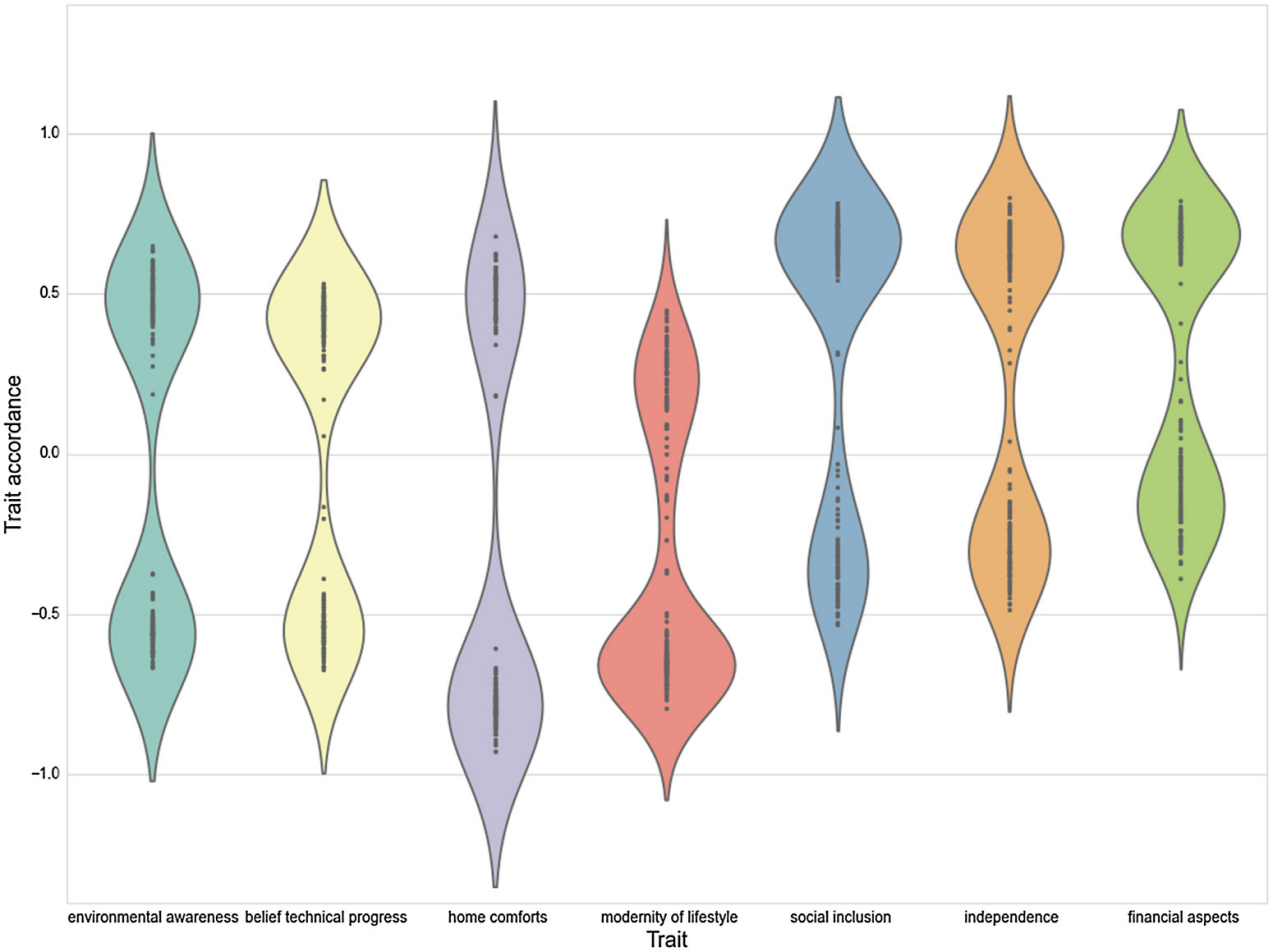
Violin plot of artificial data during simulation runs. The slower convergence process leads to separate clusters of trait accordance before convergence is reached.

The artificially generated case uses heterogeneous agents, where all trait values were randomly assigned so as to accord with those of a uniform distribution. In contrast to the empirical setting there is no fast convergence to a single cluster for every trait. The slower convergence process first leads to two separate clusters of trait accordance. This is illustrated in Fig. 5. After further steps in the simulation, levels of trait accordance become uniform, whereby the direction is heavily influenced by random selection of agents.

In general, the simulation results confirm that trait development can take different directions, depending on the initial configuration. The results from the empirical setting suggest strong path dependence. Given high homogeneity in the initial configuration, interactions will not lead to a shift of prevailing accordance/discordance with respect to specific traits. Under the artificial setting, with a lot of heterogeneity in the initial configuration, developments are much more dynamic and are driven by randomness. The outcome of early interactions heavily influences the direction in which traits develop; moreover, the emergence of separate trait clusters can be observed before convergence is reached over the long-run.

### Limitations and outlook

The diffusion model modifications outlined here are obviously subject to certain limitations. First, interaction of agents is random. However, a network topology could easily be introduced to determine the probability of interaction between two agents. In the absence of empirical interaction parameters (derived, for example, via social network analysis), a stereotypical network structure could be used as an approximation. In the current version, similarity of agents determines the strength of the influence agents exert on each other. A second limitation relates to the fact that the results of the simulations can hardly be validated against conventionally collected empirical data, as this would demand intensive data collection. This is both impracticable and unfeasible with usual sociological means (since it would demand regularly surveying the sample with regards to interactions and changes in traits). However, collecting such data by way of automated screening of online sources may seem more prospective. The ubiquity of information and communication technologies in every-day life and the advances in data mining and analysis (“Big data”) seem to give reason to further develop respective modeling options. Nonetheless, the model modification described here may still be of value. In his original work, Axelrod claimed that intuition is not a very good guide for predicting what even a simple dynamic model might produce [1]. While this is certainly true, we find that interactions based on his model reproduce expectable results, for example where (as in our empirical setting), a large degree of homogeneity prevails in the initial configuration. A further benefit of the present modification is its ability to illustrate numerous adoption scenarios, based on different, possibly empirically-based initial configurations and randomness. For the concrete case of citizen PV initiatives, the modified method could help to make stakeholders more aware of the uncertainties and random elements in diffusion processes.

## Additional information

### Axelrod’s model of cultural dissemination

Robert Axelrod suggested an agent-based model (ABM) for investigating the particularities of the dissemination of culture [1]. For this, Axelrod defined the state of an agent in terms of its cultural traits, i.e. culture was defined as consisting of a vector with F components, each with q possible values (e.g. music preferences). This vector thus $q^{F}$ spans a spectrum of an agent’s possible cultural states with size

Axelrod assumed that agents with similar traits, i.e. those with equal or similar values in certain components, will have a higher probability of influencing each other. People with no traits in common, on the other hand, will have a low probability of meeting in the first place and thus might have no influence on each other at all.^2^

**Fig. 6:**
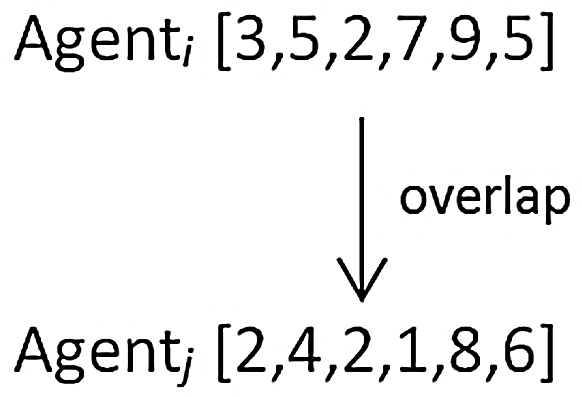
Agents as suggested by Axelrod (1997) with one “cultural” overlap in position 3 of their traits vector.

In Axelrod’s grid-based model, agents interact with each other when they possess identical traits with respect to the same components, implying that there is some form of cultural overlap (see Fig. 6).

In each iteration in the simulation agents interact with one of their von Neumann neighbors and, if this neighbor happens to have at least one trait in common, the agent will adopt another randomly chosen trait from the neighbor. The traits of those agents exhibiting overlap thus tend to produce convergence, whereas no overlap implies incommunicability. In Axelrod’s model this causes the emergence of long-lasting lock-ins of cultural affiliations which remain incapable of communicating with each other. Similar effects have been found in several variations of the Axelrod model [12], [13], [14].

### Background case: photovoltaic bottom-up initiatives

In Austria, several bottom-up initiatives in the field of collective adoption of photovoltaics (PV) have been observed in recent years [2], [3]. Citizens collectively engage in PV projects, either by investing in respective projects, or by joining initiatives that install modules on member roofs. Usually, such projects are financially more profitable than individual installations. Empirical work [15] has revealed that peer effects and social influence play a substantial role in the adoption process. Since data collection in these studies did not explicitly address social influence aspects with respect to utilization in agent-based models in mind, insights gleaned from the relevant literature were used as a proxy measure of agent interaction in order to inform an Axelrod-type simulation model. Despite this potential drawback, however, the approach taken here is rather generic in character and its application need not be confined to the specific case described above.

## Footnotes

1 Readers who are not familiar with the original model are advised to read the section “Additional Information” first.

2 No interaction occurs between agents who (already) share all their traits.
